# The role of appraisal and coping style in relation with societal participation in fatigued patients with multiple sclerosis: a cross-sectional multiple mediator analysis

**DOI:** 10.1007/s10865-016-9762-6

**Published:** 2016-07-02

**Authors:** Lizanne Eva van den Akker, Heleen Beckerman, Emma Hubertine Collette, Gijs Bleijenberg, Joost Dekker, Hans Knoop, Vincent de Groot, TREFAMS-ACE study group, V. de Groot, H. Beckerman, A. Malekzadeh, L. E. van den Akker, M. Looijmans, S. A. Sanches, J. Dekker, E. H. Collette, B. W. van Oosten, C. E. Teunissen, M. A. Blankenstein, I. C. J. M. Eijssen, M. Rietberg, M. Heine, O. Verschuren, G. Kwakkel, J. M. A. Visser-Meily, I. G. L. van de Port, E. Lindeman, L. J. M. Blikman, J. van Meeteren, J. B. J. Bussmann, H. J. Stam, R. Q. Hintzen, H. G. A. Hacking, E. L. Hoogervorst, S. T. F. M. Frequin, H. Knoop, B. A. de Jong, F. A. J. de Laat, M. C. Verhulsdonck, E. T. H. van Munster, C. J. Oosterwijk, G. J. Aarts

**Affiliations:** 1 Department of Rehabilitation Medicine, VU University Medical Center, PO Box 7057, 1007 MB Amsterdam, The Netherlands; 2 EMGO Institute for Health and Care Research, VU University Medical Center, Amsterdam, The Netherlands; 3 MS Center Amsterdam, Amsterdam, The Netherlands; 4 Department of Medical Psychology, VU University Medical Center, Amsterdam, The Netherlands; 5 Expert Centre for Chronic Fatigue, Radboud University Medical Centre, Nijmegen, The Netherlands

**Keywords:** Multiple sclerosis, Appraisal, Coping, Participation, Multiple mediator model

## Abstract

To determine the relationship between appraisal and societal participation in fatigued patients with Multiple Sclerosis (MS), and whether this relation is mediated by coping styles. 265 severely-fatigued MS patients. Appraisal, a latent construct, was created from the General Self-Efficacy Scale and the helplessness and acceptance subscales of the Illness Cognition Questionnaire. Coping styles were assessed using the Coping Inventory Stressful Situations (CISS21) and societal participation was assessed using the Impact on Participation and Autonomy. A multiple mediator model was developed and tested by structural equation modeling on cross-sectional data. We corrected for confounding by disease-related factors. Mediation was determined using a product-of-coefficients approach. A significant relationship existed between appraisal and participation (β = 0.21, 95 % CI 0.04–0.39). The pathways via coping styles were not significant. In patients with severe MS-related fatigue, appraisal and societal participation show a positive relationship that is not mediated by coping styles.

**Electronic supplementary material:**

The online version of this article (doi:10.1007/s10865-016-9762-6) contains supplementary material, which is available to authorized users.

## Introduction

MS is a progressive neurological disease, with symptoms and effects on daily life that tend to worsen over time (Compston & Coles, 2008). The most frequent symptom is severe MS-related fatigue, experienced by about 80 % of MS patients (Fox et al., 2015; Giovannoni, 2006). For many people with MS, fatigue compromises societal participation in several life domains (Compston & Coles, 2008; de Groot et al., 2008; Kierkegaard et al., 2012; Kos et al., 2008; Kwiatkowski et al., 2014) and can lead to a reduction of hours worked and early loss of employment (Induruwa et al., 2012; Kwiatkowski et al., 2014; Leocani et al., 2008).

Societal participation is defined by the World Health Organization’s International Classification of Functioning, Disability and Health (ICF) as involvement in life situations in relation to health conditions, body functions and structure, activities, and contextual factors (WHO, 2001). In a systematic review of instruments that are used to assess participation (Eyssen et al., 2011), the following working definition of societal participation was used: ‘participation is performing roles in the domains of home, family, social functioning, financial, work/education, or in a general domain’. Societal participation is an important rehabilitation outcome and it is considered to be an indicator of successful adjustment to chronic disease (WHO, 2001). Better insight into the factors that influence this rehabilitation outcome in patients with MS is a prerequisite for improved societal participation. Research has shown that disease factors such as severity of MS (Kwiatkowski et al., 2014) and poor physical functioning (Van der Hiele et al., 2014) have a negative influence on societal participation in these patients.

Disease factors alone cannot fully explain reduced societal participation (Kwiatkowski et al., 2014). Several studies have shown that psychological factors affect the societal participation of patients with chronic diseases like rheumatic disorders, cardiovascular diseases, Alzheimer’s Disease, MS, and Spinal Cord Injury (Adler & Matthews, 1994; Cameron & Leventhal, 2003; Stanton et al., 2007; Stein & Baum, 2013). Psychological factors found to be related to societal participation include successful performance of adaptive tasks, adjustment to disability, maintenance of emotional balance, the absence of psychological disorders (Maes et al., 1996), coping styles (Demers et al., 2009; Kennedy et al., 2006; Levasseur & Couture, 2015; Lindwall et al., 2012; Peter et al., 2014), and appraisal (Barnwell & Kavanagh, 1997; Peter et al., 2014).

Appraisal is the evaluation of a situation and the evaluation of one’s own abilities to deal with the situation (Lazarus & Folkman, 1987). It is a comprehensive term in which successful performance of adaptive tasks, adjustment to disability and maintenance of emotional balance can be scaled. Detailed examination of the relationships between the psychological factors that are related to societal participation suggests that the relation between appraisal and societal participation is mediated by coping processes (Lowe et al., 2008; Middleton & Craig, 2008; Peter et al., 2014). Appraisal influences the coping strategies that are used by a person (Middleton & Craig, 2008; Peter et al., 2014). Individuals actively and consciously select and engage in certain coping behaviors (Lazarus & Folkman, 1984; Parker & Endler, 1989). It appears that individuals frequently adopt certain coping preferences, and engage in particular behaviors across different situations (Endler & Parker, 1994). Consequently, the level of societal participation results from these prior coping processes (Demers et al., 2009; Kennedy et al., 2006; Levasseur & Couture, 2015; Lindwall et al., 2012; Middleton & Craig, 2008; Peter et al., 2014).

To improve societal participation in a meaningful way, we need to understand which variables determine societal participation in patients with MS-related fatigue. To the best of our knowledge, a comparable preliminary mediation analysis in patients with MS or other chronic conditions has not yet been described in the literature. Therefore, the objectives of the current study were to investigate the relationship between appraisal and societal participation in severely fatigued MS patients and to test whether this relationship is mediated by coping styles. First, we hypothesized that there is a relationship between positive appraisal and societal participation. Second, we expect that coping styles show a potential mediation effect. More specifically, we expect that patients who tend to appraise situations more negatively show more emotion or avoidance-oriented coping and subsequently perceive more problems with societal participation. Furthermore, patients with a more positive appraisal of situations would show a more task-oriented coping style, leading to greater societal participation.

## Methods

### Design

We used baseline data for individuals who were included in the Treating Fatigue in Multiple Sclerosis—Aerobic Training, Cognitive Behavioral Therapy, and Energy Conservation Management (TREFAMS-ACE) study program (Beckerman et al., 2013) (ISRCTN69520623, ISRCTN58583714 and ISRCTN82353628). The TREFAMS-ACE (Beckerman et al., 2013) program consists of three multi-center randomized clinical trials that all used the same inclusion criteria. The study was approved by the Medical Ethical Board of the VU University Medical Center Amsterdam. Patients received both written and oral information about the TREFAMS-ACE trials before providing written informed consent.

**Fig. 1:**
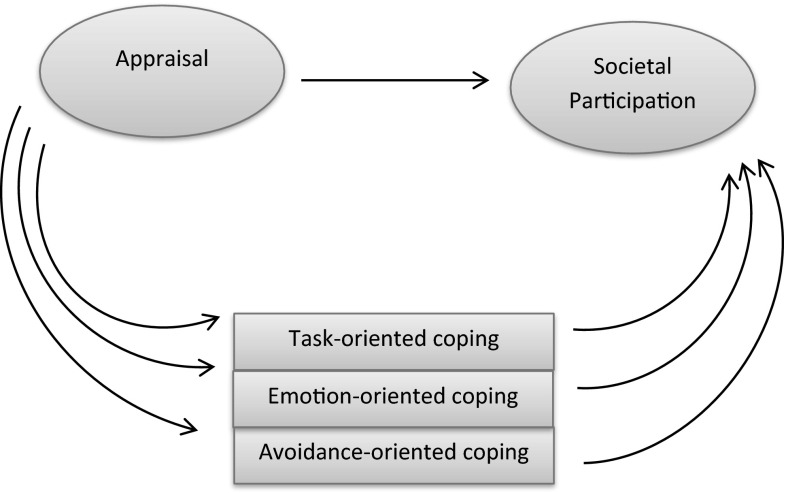
Multiple mediation model of appraisal (independent variable), coping styles (mediating variables) and societal participation (dependent variable). For clarity, the MS-related confounding factors and observed variables used for the latent variables are not displayed

A cross-sectional design was chosen to establish a basic understanding of the relationships hypothesized. Figure 1 shows the hypothesized model of appraisal (independent variable), coping styles (mediating variables) and societal participation (dependent variable). This model was adjusted for the confounding effects of MS-related disability.

### Participants

Patients were included with definite MS, experiencing severe MS fatigue (Checklist of Individual Strength fatigue subscale score ≥35) (Vercoulen et al., 1994, 1996), ambulatory (Expanded Disability Status Scale (EDSS) score ≤6.0) (Kurtzke, 1983), no signs of exacerbation, no use of a corticosteroid treatment within the past 3 months, no current infections, no anemia, and normal thyroid function. Exclusion criteria were signs of clinical depression (a score >11 on the Hospital Anxiety and Depression Scale) (Zigmond & Snaith, 1983), primary sleep disorders, severe comorbidity, a non-pharmacological treatment for fatigue in the past 3 months, a pharmacological treatment for fatigue that was started in the past 3 months, or a current/recent pregnancy.

### Measurement instruments

#### Independent variables

Demographic information was used to characterize the patients. The level of education was categorized according to National Institute of Public Health and the Environment (RIVM) guidelines as low, intermediate, or high.

Appraisal was defined as the evaluation of a situation and the evaluation of one’s own ability to deal with the situation (Lazarus & Folkman, 1984). In this study, we did not use a questionnaire to observe appraisal directly. Therefore, in order to capture the comprehensive construct ‘appraisal’, we created a latent variable. Latent constructs allow for describing relations among a class of variables that share something in common, rather than producing concrete statements that are restricted to the relation between more specific variables (Bollen, 2002). The following questionnaires were used to capture the construct appraisal, including General Self-Efficacy Scale (GSES) (Schwarzer et al., 1995) and the helplessness and acceptance subscales of the Illness Cognition Questionnaire (ICQ) (Evers et al., 1998; Evers et al., 2001).

*General Self*-*Efficacy* was assessed with the Dutch General Self Efficacy Scale (GSES) (Schwarzer et al., 1995). Self-efficacy refers to the belief that one can perform difficult tasks in various domains of human functioning by means of taking appropriate action. This entails goal-setting, persistence in face of barriers and recovery from setbacks (Schwarzer et al., 1999). The scale consists of 10 questions, which are answered on a 4-point Likert-type scale. Higher scores are related to higher self-efficacy levels. Internal consistency (Scholz et al., 2002) and convergent and discriminant validity (Schwarzer et al., 1997) are reported to be good.

*Helplessness* is measured with the Illness Cognitions Questionnaire (ICQ)-subscale helplessness. The complete questionnaire, 18 items, was developed to gain insight into the manner in which patients give meaning to their chronic disease (Evers et al., 1998). In total, three ICQ-subscales can be distinguished: helplessness, acceptance and disease benefits (Evers et al., 1998, 2001). The helplessness subscale focuses on the negative meaning patients attribute to their disease. The six helplessness questions are answered on a 4-point Likert-like scale. Whereas higher scores normally indicate increasing helplessness, for this article the scores are reversed, with higher scores indicating decreasing helplessness. The complete ICQ questionnaire is considered reliable and valid in patients with rheumatoid arthritis and MS (Evers et al., 1998, 2001).

*Acceptance* is a 6-item subscale of the ICQ, with the same scoring system as the helplessness subscale. This subscale focuses on acceptance of a negative situation, in which the negative meaning subsides. Higher scores indicate a better acceptance of the chronic disease (Evers et al., 1998, 2001).

#### Mediating variables

Coping styles were assessed with the short form of the Coping Inventory for Stressful Situations (CISS21) (Endler & Parker, 1999). Three types of coping styles are distinguished with this questionnaire: task-oriented (7 items), emotion-oriented (7 items) and avoidance-oriented (7 items) coping. Item scores range from 1 (not at all) to 5 (very strong). Task-oriented coping is also known as the problem solving coping style, i.e. targeting a stressful situation in practical ways that should consequently reduce stress (Endler & Parker, 1994). The CISS21 emotion-oriented subscale focuses on negative emotions that may result from a particular situation, i.e. blaming oneself, worrying, feeling confused, etc. Avoidance-oriented coping is about seeking other people’s company or seeking distraction (Endler & Parker, 1994). The CISS21 appears to be valid and reliable in healthy populations (De Ridder & Van Heck, 2003; Endler & Parker, 1999) and in Dutch patients with MS (De Ridder & Van Heck, 2003; Fournier et al., 1999).

#### Dependent variables

Societal participation was measured with the Impact on Participation and Autonomy questionnaire (IPA) (Cardol et al., 1999). This self-report questionnaire assesses a person’s current ability to decide how to live their life, assessing the extent to which an individual can determine when and how he or she performs activities. The questionnaire includes items such as carrying out domestic activities when one wants, and shopping and cooking the way one prefers (Cardol et al., 1999), with a total of 31 questions that can be answered on a 3, 4, or 5-point scale. In total, 5 subscales can be distinguished: 1. Autonomy indoors (5 items), 2. Family role (7 items), 3. Autonomy outdoors (7 items), 4. Social life and relations (7 items), 5. Work and education (6 items). The total average score on each IPA domain ranges from 0 to 4, formally with lower scores indicating better societal participation and autonomy. To facilitate interpretation of our study results we reversed the IPA scores, so that higher scores indicate better societal participation and autonomy. The IPA is a reliable and valid instrument for assessing societal participation in chronic medical disorders (Cardol et al., 2002).

#### Confounding variables

The following MS-related disabilities were considered for their confounding effect:

*Fatigue* was measured with the Checklist Individual Strength (CIS20r), subscale fatigue (Vercoulen et al., 1994). This subscale consists of 8 statements. Patients are asked to rate on a 7-point scale how much they agree or disagree, with fatigue scores ranging from 8 to 56 points. All patients in the present study had a score of 35 or higher before enrollment in the TREFAMS study. The CIS20r focuses on the previous 2 weeks, and is considered reliable and valid for measuring fatigue in a clinical setting in patients with MS (Vercoulen et al., 1996).

*Concentration* problems due to fatigue were measured with the subscale concentration of the CIS20r (Vercoulen et al., 1994). This subscale consists of 5 statements, with the same scoring system as the CIS20r fatigue (score range 5–35).

*Disease severity* was measured with the Expanded Disability Status Scale (EDSS), which was determined by a trained rehabilitation physician (Kurtzke, 1983). The EDSS score ranges from 0 to 10, with lower scores representing lower disease severity. An EDSS score ≤6.0 was used as an inclusion criterion only to ensure selection of ambulatory patients (Beckerman et al., 2013).

*Physical functioning* was measured with the SF36-physical functioning (Aaronson et al., 1998). This subscale consists of 10 questions, scored on a 3-point scale: 1 = restricted a lot (0 points), 2 = restricted a little (50 points) and 3 = not restricted at all (100 points). The total score is derived by calculating the average score. The total score ranges from 0 to 100, with a higher score representing better physical functioning (Aaronson et al., 1998). The SF36-physical functioning is suitable for measuring physical functioning in patients with MS (de Groot et al., 2006).

*Mental health* was measured with the SF36 subscale mental health (Aaronson et al., 1998). This subscale consists of 5 questions, with the same scoring system as the SF36-physical functioning.

### Statistical analysis

The demographics of the study population were analyzed using SPSS 20 for windows (SPSS Inc., Chicaco, IL). Normality assumptions of the individual variables were checked by visual inspection of histograms and normal probability plots (Weston et al., 2008).

Mplus (version 6.1) was used for structural equation modeling (SEM). SEM is valid for samples with more 200 participants (Weston et al., 2008). This statistical technique was preferred due to its ability to measure underlying hypothetical constructs and their interrelations (Tomarken & Waller, 2005; Weston et al., 2008). Both so-called observed and constructed latent variables can be used for SEM.

The hypothesized model tested with SEM in this study is presented in Fig. 1. The model consists of one independent latent construct for appraisal, one dependent latent construct for societal participation, three mediating coping styles, and one set of confounding variables. First, the latent variables *appraisal* and *participation* were constructed (step 1). When the observed variables contributed significantly to the constructed latent variables they were maintained in the model. Second, we tested the relationship between the latent construct appraisal and the latent outcome societal participation (step 2). In step 3, we included the set of potentially confounding variables that measure MS-related disability in the model. If the relationship between appraisal and participation changed more than 10 %, relevant confounding was present (Bouter et al., 2010) and the set of variables was maintained in the model. Step 4 resulted in an adjusted relationship between appraisal and participation. In step 5 we used the product-of-coefficients approach of (MacKinnon et al., 2002, 2004), to determine whether coping styles acted as mediators in the relation between appraisal and participation. Mediation is confirmed (step 6) if appraisal has a significant direct effect on participation and a significant indirect effect on participation. To study the indirect effect, the two indirect pathways (step 5a and step 5b) are multiplied: i.e. the pathway from appraisal to coping style was multiplied with the pathway from coping style to participation. This was performed separately for the three coping styles. In order to correct for confounding, the mediators (coping styles) in the model were also corrected for MS-related disabilities (Hayes, 2013). To determine the Confidence Intervals (CI’s) and the significance of mediation (step 5 and step 6), we performed a bootstrap (data re-sampling) procedure with 5000 bootstrap re-samples; literature indicates that 5000 re-samples is enough (Hayes & Scharkow, 2013; MacKinnon et al., 2002; MacKinnon, 2008; Preacher & Hayes, 2008; Taylor & MacKinnon, 2012). This is a more valid and powerful method for testing mediation effects than the (Sobel, 1982) test or the ‘causal steps approach’ (Baron & Kenny, 1986).

## Results

A total of 265 patients were included in the analysis (67 male, 198 female), with a mean age of 46.7 years (range 20–68), and the majority suffered from relapsing remitting MS (n = 190). The median EDSS score was 2.5 (range 0–6) and time since diagnosis was 6.6 years (range 0.1–30.7). See Table 1 for a summary of all patient characteristics. Table 2 shows the mean scores, the observed range, possible ranges and interpretation for all variables included in the analyses. The mean CIS20r-fatigue score was 43.4 (SD 7.6), with a possible range of 8–56. The mean SF36-Physical functioning score was 59.0 (SD 23.9) and the mean SF36-Mental health score was 67.1 (SD 13.2), with a possible range of 0–100. Visual inspection of histograms and normal probability plots revealed that all variables were normally distributed.

**Table 1:** Socio-demographic and disease-related characteristics of 265 patients with MS

Characteristic	n	**%**
Gender		
Male	67	25.3
Female	198	74.7
Age in years (mean, SD)	46.7	10.5
Type of MS		
Relapsing remitting	190	71.7
Primary progressive	24	9.1
Secondary progressive	33	12.5
Unknown	18	6.8
Level of education*		
Low	138	52.1
Medium	102	38.5
High	23	8.7
Unknown	2	0.8
Living situation		
Living with partner	205	77.4
Living without partner	60	22.6
Employment status		
Full-time	28	10.6
Part-time	99	37.4
Disability pension	46	46.5
Unemployed	110	41.5
Disability pension	88	80
(Early) retirement	14	5.3
Study	11	4.2
Unknown	3	1.1

* Categories for level of education were determined using the National Institute of Public Health and the Environment (RIVM) guidelines

**Table 2:** Mean (SD) scores, ranges, possible ranges and interpretation of the variables included in the theoretical model

Questionnaire	n	Mean (SD)	Range	Possible range	Interpretation
Constructs					
*Appraisal*					
GSES	n = 262	30.5 (4.7)	11–40	10–40	Higher values indicate more self-efficacy
ICQ-helplessness	n = 263	16.9 (3.3)	9–23	6–24	Higher values indicate less helplessness^a^
ICQ-acceptation	n = 263	15.1 (3.8)	6–24	6–24	Higher values indicate more acceptation
*Participation*					
IPA autonomy indoors	n = 263	3.2 (0.6)	1–4	0–4	Higher values indicate better participation^a^
IPA family role	n = 263	2.5 (0.7)	0.7–4	0–4	
IPA autonomy outdoors	n = 263	2.3 (0.7)	0.4–4	0–4	
IPA social life and relationships	n = 263	3.0 (0.5)	1.1–4	0–4	
IPA work and education	n = 253	2.1 (0.8)	0–4	0–4	
*Confounding variables*					
CIS20r-fatigue	n = 264	43.4 (7.6)	14–56^b^	8–56	Higher values indicate more fatigue
CIS20r-concentration	n = 264	20.9 (7.6)	5–35	5–35	Higher values indicate more concentration problems
EDSS median, (ICQ_1_–ICQ_3_)	n = 255	2.5 (2.0–3.5)	0–6^c^	0–10	Higher scores indicate more disease severity
SF36-physical functioning	n = 265	59.0 (23.9)	0–100	0–100	Higher values indicate better physical functioning
SF36-mental health	n = 262	67.1 (13.2)	24–92	0–100	Higher values indicate better mental health
*Mediating coping styles*					
CISS21 task-oriented	n = 263	24.3 (4.8)	7–35	7–35	Higher values indicate a stronger tendency to a certain coping style
CISS21 emotion-oriented	n = 263	18.0 (6.1)	7–31	7–35	
CISS21 avoidance-oriented	n = 264	18.7 (4.8)	7–31	7–35	

CIS20r, Checklist Individual Strength; CISS21, Coping Inventory Stressful Situations; EDSS, Expanded Disability Status Scale; GSES, General Self-Efficacy Scale; ICQ, Illness Cognition Questionnaire; ICQ_1–3_, Interquartile; IPA, Impact on Participation and Autonomy; SF36, Short Form 36

^a^Scores are reversed for interpretation

^b^CIS20r fatigue enrollment scores differed incidentally from baseline scores used in this article; CIS20r-fatigue = 14 appeared in 1 participant

^c^Used as an inclusion criterion; EDSS 0–6

### Multiple Mediator Model

**Fig. 2:**
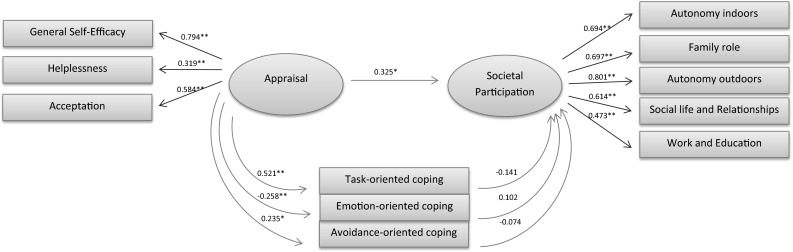
Final Multiple Mediator Model: relation of appraisal and societal participation, mediated with task-oriented, emotion-oriented and avoidance-oriented coping style. Adjustments for confounding by MS-related disability are not presented in the figure. *p ≤ 0.05; **p = 0.001

Table 3 and Fig. 2 show the results of the SEM (step 1 to step 6). The standardized factor loadings of the questionnaires (observed variables) that were used to capture the constructed latent variables *appraisal* and *participation* are all statistically significant and were retained in the model (step 1), which indicates that the two created latent variables represent accurate constructs. Adding the set of potentially confounding variables led to a more than 10 % change of the coefficient between appraisal and participation [β from 0.52 to 0.21 (R^2^ from 0.27 to 0.37)], and was thus retained in the model (step 2 and step 3); in both steps a significant relationship existed between appraisal and societal participation.

**Table 3:** Results per step of the mediation analyses

		Construct latent variables	R^2^	Adding confounders	R^2^	Adding mediators	R^2^
		β (95 %CI)		β (95 %CI)		β (95 %CI)	
1. Constructed latent variables							
Appraisal^a^	GSES	0.59 (0.47; 0.71)		0.61 (0.48; 0.73)		0.64 (0.55; 0.74)	
	ICQ-helplessness	0.65 (0.53; 0.77)		0.55 (0.43; 0.68)		0.58 (0.48; 0.68)	
	ICQ-acceptation	0.67 (0.55; 0.79)		0.74 (0.62; 0.87)		0.62 (0.53; 0.72)	
Participation^a^	Autonomy indoors	0.68 (0.60; 0.77)		0.70 (0.62; 0.78)		0.70 (0.62; 0.77)	
	Family role	0.70 (0.62; 0.78)		0.70 (0.62; 0.78)		0.70 (0.63; 0.78)	
	Autonomy outdoors	0.82 (0.76; 0.89)		0.79 (0.73; 0.86)		0.80 (0.74; 0.87)	
	Social life and relationships	0.65 (0.56; 0.73)		0.61 (0.52; 0.70)		0.62 (0.54; 0.71)	
	Work and education	0.50 (0.39; 0.60)		0.47 (0.36; 0.39)		0.49 (0.38; 0.59)	
2. Relation appraisal and participation		*0.52* (*0.38; 0.67*)	0.27				
3. Adjustment for confounders^b^							
Confounding on the relation of appraisal and participation	CIS20r-fatigue			−0.18 (−0.31; −0.06)		−0.12 (−0.25; 0.01)	
	CIS20r-concentration			−0.17 (−0.30; −0.05)		−0.15 (−0.27; −0.02)	
	EDSS			−0.06 (−0.22; 0.09)		−0.04 (−0.19; 0.10)	
	SF36-physical functioning			0.37 (0.21; 0.53)		0.32 (0.16; 0.48)	
	SF36-mental health			0.10 (−0.04; 0.23)		−0.01 (−0.20; 0.18)	
4. Adjusted relation appraisal and participation				*0.21* (*0.04; 0.39*)	0.37		
5. Multiple mediation							
5a. Appraisal-coping	Task-oriented					0.39 (0.40; 0.85)	
	Emotion-oriented					−0.63 (−1.74; −0.93)	
	Avoidance-oriented					−0.11 (−0.52; −0.08)	
5b. Coping- participation	Task-oriented					−0.13 (−0.03; 0.002)	
	Emotion-oriented					−0.63 (−0.002; 0.04)	
	Avoidance-oriented					−0.11 (−0.01; 0.01)	
5a * 5b Indirect relations	Appraisal-task-oriented coping-participation					−0.08 (−0.17; 0.01)	
	Appraisal-emotion-oriented coping-participation					−0.03 (−0.40; 0.33)	
	Appraisal-avoidance-oriented coping-participation					−0.01 (−0.99; 0.98)	
6. Final result multiple mediation model							
Total indirect						−0.12 (−0.20; 0.03)	
Direct relation appraisal and participation						*0.35* (*0.12; 0.57*)	0.40

*CI* confidence interval

^a^Results are standardized factor loadings

^b^The relations of the five confounders with the three coping styles (i.e. 15 relations) are not displayed for clarity

After examining the adjusted relation between appraisal and participation, we added the mediators and, using a product-of-coefficients approach, determined with whether relevant mediation occurred. The results of the analyses (steps 5a, 5b and 6) confirmed the first criterion of mediation: i.e. a significant relation between appraisal and participation [β = 0.35, 95 % CI 0.12–0.57 (R^2^ 0.40)]. The second criterion was not confirmed: the separate indirect effects via task-oriented (β = −0.08, 95 % CI −0.17 to 0.01), emotion-oriented (β = −0.03, 95 % CI −0.40 to 0.33) and avoidance-oriented (β = −0.01, 95 % CI −0.99 to 0.98) were not statistically significant. Furthermore, the total indirect effect was also not statistically significant (β = −0.12, 95 % CI −0.20 to 0.03). Therefore, coping styles do not mediate the relation between appraisal and societal participation, an outcome in conflict with our second hypothesis.

## Discussion

This study showed a robust relationship between appraisal and societal participation, a result that supports our first hypothesis, previous research in patients with MS (Barnwell & Kavanagh, 1997) and research in patients with spinal cord injury (Peter et al., 2014). A positive view of situations and the ability to deal with them is related to better societal participation. Unexpectedly, our results showed that coping styles did not mediate this relationship.

Even though the total mediation pathway did not fulfill the criteria for mediation, significant relationships were found between appraisal and coping styles (step 5a). Positive appraisal is related to a task-oriented coping style, while negative appraisal of situations is related to an emotion-oriented coping style, as was hypothesized. Contrary to our expectations and those of others (Peter et al., 2014; Tan-Kristanto & Kiropoulos, 2015), we found a positive relation between appraisal and avoidance-oriented coping. This means that appraising situations positively can lead to avoidance behavior. A possible explanation for this outcome may lie in our use of the CISS21 questionnaire to measure avoidance-oriented coping. The items in this questionnaire relate to distraction-seeking rather than conscious avoidance of a situation (Endler & Parker, 1994). For patients who appraise situations positively it may temporarily suffice to seek distraction through such activities as calling/visiting friends or self-pampering, e.g. when a task-oriented approach is not possible. This strategy may offer short-term relief and may be useful in certain situations (Endler & Parker, 1994). In addition, it should be kept in mind that different coping styles can co-exist; high scores on one type of coping does not mean that one cannot achieve high scores on other types of coping. We believe that a more positive coping style relates to a patient’s capabilities and the flexibility to switch between suitable coping styles in different situations.

In the current study no relationship was found between coping styles (separate and total; step 5b and step 6) and societal participation. Likewise, Peter et al. (2014) found no support for the contribution of coping styles to societal participation in patients with spinal cord injury, except for humor as a positive reframing coping style. Therefore, in the absence of mediation by coping styles, societal participation depends on processes other than coping. An example is illustrated by the confounding results of this study: a set of the following confounders had a significant influence on societal participation: fatigue, concentration, disease severity, physical functioning and mental health.

The key strength of this study was the use of SEM to study the hypothetical mediation model. By using SEM we were able to create latent constructs and to assess the presence of mediation per coping style and for coping as a whole. SEM is gaining in popularity due to these abilities (Tomarken & Waller, 2005; Weston et al., 2008). Furthermore, by using SEM we were able to assess the presence of mediation per coping style and for coping as a whole. In addition to SEM, we used bootstrapping to improve the precision of the estimates (Hayes, 2013).

Some limitations should be considered when interpreting our results. This study was exploratory in nature, and used a cross-sectional design, which means that no conclusions can be drawn about causality (Maxwell & Cole, 2007). Future longitudinal research should be conducted to study the plausible causal relationship between appraisal, coping, and societal participation. Emotion-oriented coping includes managing emotions evoked by a stressful situation (Endler & Parker, 1990). However, the CISS21 emotion-oriented subscale focuses on the negative emotions that a situation conveys, i.e. blaming oneself, worrying, feeling confused, etc. (Endler & Parker, 1994). Likewise, caution is warranted when interpreting the IPA results. The IPA was originally used to measure participation and autonomy (Cardol et al., 1999). However, this questionnaire focuses more on autonomy, i.e. whether patients can decide how and when societal participation activities take place, rather than on the (true) amount of social activities.

Despite these limitations, this study might have the following clinical implications. This study showed that in fatigued patients with MS, the construct of appraisal plays a role in societal participation. In clinical practice, one may want to emphasize the importance of increasing self-efficacy and disease acceptance, and decreasing helplessness. A therapy that might assist in improving appraisal is Cognitive Behavioral Therapy (CBT). CBT challenges thoughts, stimulates more positive appraisal and improves confidence when dealing with situations (van Kessel et al., 2008). The close link between appraisal and societal participation has also been demonstrated in other chronic diseases in which positive self-efficacy appears to be associated with greater societal participation (Geyh et al., 2012; Lowe et al., 2008; van der Slot et al., 2010). Our results also support a relationship between appraisal and coping, and due to the cross-sectional design, it can also be argued that coping influences appraisal. Future longitudinal research should examine this potential causal relationship.

## Conclusion

In patients with severe MS-related fatigue, appraisal and societal participation show a positive relationship that is not mediated by coping styles. Future longitudinal research will be required to draw conclusions regarding causality.

## Electronic supplementary material

Below is the link to the electronic supplementary material.

Supplementary material 1 (DOCX 46 kb)
