# Supplementary material for: The role of appraisal and coping style in relation with societal participation in fatigued patients with multiple sclerosis: a cross-sectional multiple mediator analysis
Source: J Behav Med. 2016 Jul 2;39(5):855–65. doi: 10.1007/s10865-016-9762-6 (PMC5012251; doi:10.1007/s10865-016-9762-6)
Supplement: Supplementary file 1 — Supplementary material 1 (DOCX 46 kb) [file 10865_2016_9762_MOESM1_ESM.docx]

**The Role of Appraisal and Coping style in relation with Societal Participation in Fatigued Patients with Multiple Sclerosis: A Cross-sectional Multiple Mediator Analysis**

Lizanne Eva van den Akker, Heleen Beckerman, Emma Hubertine Collette, Gijs Bleijenberg^5^, Joost Dekker, Hans Knoop, Vincent de Groot, and [TREFAMS-ACE study group](http://www.ncbi.nlm.nih.gov/pubmed/?term=TREFAMS-ACE%20study%20group%5BCorporate%20Author%5D)¶

**Supplementary material:**

Emotion-oriented coping

Avoidance-oriented coping

Task-oriented coping

Concentration

EDSS

Physical functioning

Mental health

Fatigue

Multiple mediation model of appraisal (independent variable), coping styles (mediating variables), societal participation (dependent variable) and the confounders
